# Health effects of dietary phospholipids

**DOI:** 10.1186/1476-511X-11-3

**Published:** 2012-01-05

**Authors:** Daniela Küllenberg, Lenka A Taylor, Michael Schneider, Ulrich Massing

**Affiliations:** 1 Tumor Biology Center, Dept. of Clinical Research, Breisacher Straße 117, D-79106 Freiburg, Germany; 2 University Hospital Heidelberg, Pharmacy, Im Neuenheimer Feld 670, D-69126 Heidelberg, Germany; 3 Lecithos Consulting, Meyers Land 12, D-21266 Jesteburg, Germany

**Keywords:** Phospholipid, glycerophospholipid, therapy, plasma membrane

## Abstract

Beneficial effects of dietary phospholipids (PLs) have been mentioned since the early 1900's in relation to different illnesses and symptoms, e.g. coronary heart disease, inflammation or cancer. This article gives a summary of the most common therapeutic uses of dietary PLs to provide an overview of their approved and proposed benefits; and to identify further investigational needs.

From the majority of the studies it became evident that dietary PLs have a positive impact in several diseases, apparently without severe side effects. Furthermore, they were shown to reduce side effects of some drugs. Both effects can partially be explained by the fact that PL are highly effective in delivering their fatty acid (FA) residues for incorporation into the membranes of cells involved in different diseases, e.g. immune or cancer cells. The altered membrane composition is assumed to have effects on the activity of membrane proteins (e.g. receptors) by affecting the microstructure of membranes and, therefore, the characteristics of the cellular membrane, e.g. of lipid rafts, or by influencing the biosynthesis of FA derived lipid second messengers. However, since the FAs originally bound to the applied PLs are increased in the cellular membrane after their consumption or supplementation, the FA composition of the PL and thus the type of PL is crucial for its effect. Here, we have reviewed the effects of PL from soy, egg yolk, milk and marine sources. Most studies have been performed *in vitro *or in animals and only limited evidence is available for the benefit of PL supplementation in humans. More research is needed to understand the impact of PL supplementation and confirm its health benefits.

## Introduction

**Figure 1:**
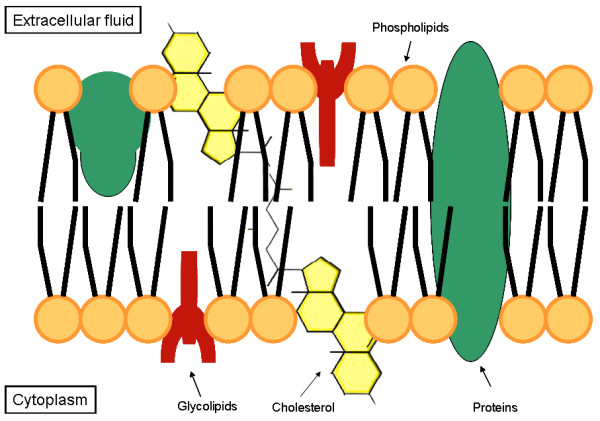
**Schematic illustration of the plasma membrane of an eukaryotic cell**.

Phospholipids (PLs) are amphiphilic lipids found in all plant and animal cell membranes, arranged as lipid bilayers (Figure 1). The PLs found in most cell membranes are basically glycerophospholipids (GPLs), which consist of fatty acids (FAs) esterified to a glycerol backbone, a phosphate group and a hydrophilic residue (e.g. choline, resulting in phosphatidylcholine or lecithin). The backbone of a PL can also be the long chain amino-alcohol sphingosin instead of glycerol. These PL are classified as sphingophospholipids, the most representative being sphingomyelin, found in high quantities in brain and neural tissue, consisting of sphingosin esterified to one FA and phosphocholine.

Figure 1 shows a simplified illustration of a typical eukaryotic cellular plasma membrane. Besides glycerophospholipids (GPLs) and sphingomyelin (SPM), biological membranes are also made up of glycolipids and cholesterol, as well as of integral and peripheral membrane proteins. Most of the GPLs arrange themselves forming a lipid bilayer, in which the polar (hydrophilic) regions of the PL are directed towards the outer surface of the membranes and the hydrophobic regions towards the inner membrane compartment.

GPLs extracted from food products (e.g. soybeans, egg yolk, milk, or marine organisms like fish, roe or krill) are defined as "dietary GPLs". They can be ingested either with normal diet or as supplements. Naturally occurring GPLs, either from plant or animal origin, predominantly contain an unsaturated FA in the *sn*-2 position^1^, such as oleic, linoleic or linolenic acid, or the proinflammatory arachidonic acid (usually from animal origin) or the anti-inflammatory eicosapentaenoic acid (usually from marine origin), while the *sn*-1 position predominantly carries a saturated FA, such as stearic acid or palmitic acid.

The mean dietary intake of GPLs is not exactly known. In a normal diet, the daily intake of PC is approximately 2-8 grams [1]. Foods with a high PC content are e.g. egg yolk, pig or chicken liver, soybeans and beef.

In the intestine, GPLs are almost completely absorbed (> 90%). In the lumen most of them are hydrolysed at the *sn*-2 position by the pancreatic phospholipase A_2 _(pPLA_2_) and then taken up by the enterocytes as free fatty acids (FFAs) and lysoPL. Both can be reesterified to GPLs and enter the bloodstream incorporated in chylomicrons and, in a small proportion, in very low density lipoproteins (VLDL). However, it has been assumed that almost 20% of intestinal PLs are absorbed passively and without hydrolysation [2], and preferentially incorporated directly into high density lipoproteins (HDL). From HDL, GPLs can be transferred into the plasma membranes of numerous cells (e.g. liver, muscle, kidneys, lung, tumor cells, etc.) as their corresponding lyso-form after enzymatic activity of the lecithin-cholesterol-acyl-tranferase (LCAT) [3]. This mechanism is complex and has not yet been completely elucidated, but it has been shown that dietary GPLs are able to deliver their FAs for incorporation into cellular membranes, thus altering the membrane composition of the cells [4].

Since GPLs are the main PL class found in cell membranes, their FA composition has a major impact on membrane characteristics, for example membrane fluidity and therefore formation of lipid rafts. Lipid rafts are dynamic membrane micro-domains with a high content of cholesterol and PLs predominantly carrying saturated FAs and have been involved in apoptosis and cellular proliferation [5]. These micro-domains are in charge of organising and regulating important cellular functions. An important example is the lipid raft dependent trimerisation of Fas-receptor (CD95), pivotal for the induction of apoptosis after Fas-binding. Furthermore, PLs play an important role in providing unsaturated FAs as precursors for eicosanoid synthesis. The origins of the FA, therefore their chain length and degree of saturation, contribute to the biological properties of the synthesized eicosanoids. Depending on which PL-bound FAs are available in the cell membrane, different types of eicosanoids are synthesized (e.g. the proinflammatory PGE2 synthesized from arachidonic acid and the predominantly anti-inflammatory PGE3 from eicosapentaenoic acid) [6].

In summary, the oral application of dietary GPLs with a specific FA composition has the potential to cause defined alterations of the FA composition of membrane PLs within a certain cell type. As a consequence, cellular functions, including signaling and transport, as well as the activity of membrane bound enzymes, could be modulated by dietary PLs and hence contribute to the health benefits described throughout this review article.

Different types of dietary GPLs vary in their FA-composition and headgroup, and therefore may have different effects. Table 1 provides an overview of the regular composition of dietary GPLs; and table 2 provides a selection of PL products used as supplements in the published papers included in this review.

**Table 1:** Important dietary glycerophospholipids (GPLs): PL and FA composition

		Soybean GPL *	Egg yolk GPL*	Milk GPL *	Marine GPL *
**PL's^+^**	PC	10-15% [111]	65-70% [111]	26% [110]	87.5%^§^

	PE	9-12% [111]	9-13% [111]	30% [110]	5.8%^§^

	PI	8-10% [111]	--	9% [110]	2.2%^§^

	PS	1-2% [111]	--	--	--

	SPM	--	2-3% [111]	22% [110]	3.4%^§^

**Unsaturated Fatty acids**	Total	75.5% [111]	54% [111]	28% [M.S. unpublished Data]	84.3%^§^

	Oleic	10.7% [111]	32.3% [111]	20% [M.S. unpublished Data]	29.2%^§^

	Linoleic	58% [111]	16.7% [111]	2.2% [M.S. unpublished Data]	2.5%^§^

	Linolenic	6.8% [111]	--	0.5% [M.S. unpublished Data]	2.7% [L.T. unpublished Data]

	Arachidonic	--	5% [111]	0.1% [M.S. unpublished Data]	1.9% [L.T. unpublished Data]

	eicosapentaenoic	--	--	--	18.8%^§^

	docosahexaenoic	--	--	--	22.8%^§^

	other	--	--	5% [M.S. unpublished Data]	--

**Saturated** **Fatty Acids**	Total	22.4% [111]	46% [111]	68.7% [M.S. unpublished Data]	15.6%^§^

	Palmitic	18.4% [111]	37% [111]	31.8% [M.S. unpublished Data]	14.1% [L.T. unpublished Data]

	Stearic	4% [111]	9% [111]	15% [M.S. unpublished Data]	2.9% [L.T. unpublished Data]

	other	--	^--^	C4:0 to C14:0 24.1% [M.S. unpublished Data]	--

* Mean values of different analytical data. The table contains the typical composition of the referred GPL, which may differ depending on its source and the employed analytical method. Information was extracted from the mentioned literature or from the analytical data sheets of the manufacturers.

^+ ^Phosphatidylserine (PS) is also an important GPL found mostly in meat. A good source is bovine brain. This compound is not mentioned here, since it is not widely addressed in the present review.

^§ ^Data from BioSea Management AS.

**Table 2:** Selected examples of dietary glycerophospholipids (GPLs) used as supplements in the mentioned studies

Brand name/Product name	Product information given by the producer/publication
**Dietary GPLs from soybean**	

Essentiale^®^	80% PC, other ingredients: soybean oil, castor oil, ethanol, ethylvanillin, methoxyacetophenon, food colourings, gelatine, tocopherol and water; as capsules. Nattermann & Cie. GmbH

Essentiale L^® ^or Buer^® ^Lecithin	Soybean GPL (purity unknown), other ingredients: riboflavin, phosphate sodium, pyridoxine hydrochloride, cyanocobalamin, sodium panthotenate and nicotinamide; as capsules. Nicholas Piramal India Ltd. Or Nycomed Germany GmbH

Lipoid S45^®^	45-50% soybean PC, 10-18% PE, max. 4% LysoPC. Typical FA composition: 58-65% linoleic acid, 12-17% palmitic acid, 8-12% oleic acid and other FAs; as powdered compound. Lipoid GmbH

Lipostabil^®^	93% PC, other ingredients: ethanol, benzyl alcohol, deoxycholic acid, sodium hydroxide, sodium chloride, tocopherol and water; as liquid formulation. Artegodan GmbH

PC-55	55% PC, 30% PE, 3% PI, other PLs and triglycerides; as powdered compound. TwinLab [77]

PhosChol	80% soybean lecithin (Phosal 75A), 18% TG, other ingredients: anethole, tocopherol and paraben. Fatty acid composition: mainly linoleic acid; as liquid formulation. Nutrasal LLC Co. [66]

Phospholipon^®^100	100% native soybean PC as a powdered compound. Phospholipid GmbH

Phospholipon^®^90G	94-100% PC, LysoPC and tocopherol as powdered compound. Phospholipid GmbH

Polyunsaturated PC	PC with 40-52% linoleic acid (n-6); as powdered compound. Rhone-Poulenc Rorer GmbH [104]

Polyunsaturated Lecithin	60% PC, 30% PE, 6% phosphatidic acid, 3% monophosphatidylinositol, 3% LysoPC, 80% unsaturated fatty acids, 20% other fatty acids; as powdered compound. American Lecithin Co. [102]

Soy Lecithin	31.7% PC, 20.8% PE, 3% PS, 17.5% PI, 2% phosphatidic acid, other ingredients unknown; as powdered compound. Herbarium, Brazil [99]

Soy Lecithin	40.4% PC, 35.1% PE, 24.5% PI. Fatty acid composition: 56.6% linoleate, 18.9% palmitate and other fatty acids; as powdered compound. Central Soya, Fort Wayne IN [53]

Soybean PC	96.5% PC as powdered compound. Tsujiseiyu Matsuzaka Co. [29]

Soybean PC capsules	64% soybean PC, 30% soybean oil, other PLs, ethanol and water. Fatty acid composition: 64% linoleic acid, 12% palmitic acid, 12% oleic acid, 8% linolenic acid and 4% stearic acid; as liquid compound. Nutrition et Santé, Revel, France [54]

Sterpur P-30	30% PC, 21% PE and 8% PI, other ingredients unknown; as granulate. Stern-Lecithin & Soya GmbH [39,112]

**Dietary GPLs from milk**	

Lacprodan^® ^PL-20	27% PC, 22% PE, 8% PI, 27% SPM, 12% PS and other ingredients; as powdered compound. Arla Foods Ingredients [57]

Sphingomyelin	Extracted from bovine milk, unknown composition, as powdered compound. Avanti Polar Lipids, Inc. [34]

**Dietary GPLs from marine origin**	

MPL (Neptune Krill oil, NKO™)	40% PL, 15% EPA and 9% DHA bound to PLs and neutral lipids, other ingredients: unsaturated FAs, saturated FAs, gelatine, glycerine, water; as liquid formulation packed in gelatine capsules. Jarrow Formulas Co.

MPL (Vitalipin^®^)	29% PC, 18% EPA and 26% DHA bound to PLs and neutral lipids, other ingredients: polyenes, monounsaturated FAs, saturated FAs, other fats; as liquid formulation packed in gelatine capsules. Membramed health food GmbH

Squid meal PC	PC contains 35.2% palmitic acid, 9.2% EPA, 43% DHA and 13% other FAs; as powdered compounds. Nippon chemical Feed Co. Ltd. [30]

**Dietary GPLs from animal origin**	

Bovine liver PI	100% PI as powdered compound. Avanti Polar Lipids, Inc. [61]

Brain cortex derived PS	Unknown composition. Fidia Farmaceutical S.p.A. [89]

Pig brain GPLs	PLs with aprox. 23% saturated fatty acids, 53% monounsaturated fatty acids and 24% n-6 fatty acids, as powdered compound. Laboratoires Ponroy, France [71]

The main characteristics of dietary GPL are:

◦ Soybean GPLs are mainly characterised by their high content of unsaturated FAs, namely linoleic acid (n-6 FA) bound to different types of PLs. The relative amounts of the PL classes PC, phosphatidylethanolamine (PE) and phosphatidylinositol (PI) are similar.

◦ Main PL class of egg yolk is PC. The FA distribution represents mainly unsaturated FAs, in particular oleic acid.

◦ Milk GPLs are not only characterised by having PC and PE as main PL classes, but also by containing high quantities of SPM. Bound FAs are both saturated and unsaturated.

◦ The main PL class of marine-derived GPLs is PC, predominantly binding the unsaturated eicosapentaenoic acid (EPA) and docosahexaenoic acid (DHA); both are n-3 FAs.

On the cellular level, the effects of membrane modifications caused by PLs are easy to detect by *in vitro *analysis. In contrast, membrane alterations are difficult to determine in vivo and especially in humans. Until now, the gastrointestinal metabolism of dietary GPLs as well as the mechanism of their incorporation into cellular membranes to achieve beneficial health effects has not been thoroughly explored. One main and obvious difficulty for these kinds of studies is that GPLs are normally found in almost all kinds of food products. The different types of GPLs can not be separated within each food product and at the same time other lipid components like triglycerides (TGs) are also included. This makes the determination of the metabolic pathway of each GPL difficult to analyse and thus their health effects can not be clearly defined.

This review article summarises the published health effects of dietary GPLs for different groups of illnesses. Most of these nutritional studies have focussed only on the health effects of GPLs, but, like mentioned before, the underlying physiological mechanisms have not been addressed. However, a few studies have shown that the FA composition of cellular membranes can be influenced by dietary GPLs. Around 100 articles dealing with the effects of dietary PLs were reviewed, including human clinical trials as well as *in vitro *and animal studies.

### Phospholipids in inflammatory processes

Inflammation is a biological response to harmful stimuli, such as pathogens or damaged cells, which is induced and kept up by a biochemical cascade involving inflammatory mediators like cytokines, chemokines and eicosanoids. There is a growing scientific rationale for the use of dietary PLs for the treatment of inflammatory diseases to regulate the inflammatory reaction.

Hartmann et al. investigated the effect of PC (unknown source) in chemically-induced arthritis (with carrageen) in rats. They found a significantly reduced development of arthritis after PC supplementation, most likely due to an inhibition of the neutrophil leukocyte-mediated inflammatory reaction [7]. Further, Eros et al. could show that soybean PC (Lipoid S45, see table 2) alone can limit the inflammatory process of joints in a chronic murine model of rheumatoid arthritis (collagen-induced arthritis) when fed during the onset of disease [8]. They found oral pretreatment of mice with soy PC to effectively impair leukocyte adhesion to the endothelial layer as well as to decrease the expression of inducible Nitric Oxide Synthase (iNOS) and thereby decrease the degree of synovial angiogenesis. In another study they investigated the same dietary supplementation of soybean PC (Lipoid S45, see table 2) in mice after inducing pleurisy. Inflammatory reaction was characterised by pulmonary leukocyte infiltration, mast cell degranulation and increased iNOS, all parameters were suppressed by dietary soybean PC [9]. Soybean PC was therefore shown to be effective in reducing inflammatory reactions in arthritis and similar inflammatory processes in a murine model.

Prostanoids play a central role in inflammatory processes and pain. The isoenzymes cyclooxygenase (COX)-1 and -2 are responsible for the oxygenation of two essential FAs, dihomo-gamma-linolenic acid (DGLA, a n-6 FA) and EPA (a n-3 FA), resulting in prostanoids of the series-1 and series-3, which are less inflammatory than those of series-2, which result from the oxygenation of arachidonic acid (AA, a n-6 FA). Since DGLA and EPA are competitive inhibitors of AA for enzymatic COX conversion, they are responsible for reducing inflammatory properties, by decreasing the enzymatic conversion of prostanoids of the series-2. Dietary sources of DGLA and EPA (e.g. borage or fish oil) could therefore alleviate inflammatory reactions.

Marine phospholipids (MPL), carrying mainly n-3 FAs, have been described to reduce inflammatory reactions by the inhibition of prostaglandins of the series-2 (PGE2). Krill oil (see table 2), a PC rich marine product extracted from an antarctic zooplankton crustacean was investigated for its anti-inflammatory effects in patients with cardiovascular and/or rheumatoid arthritis and elevated C-reactive protein (CRP) levels in a randomised, placebo-controlled, double-blind, manufacturer-sponsored study. Treatment with krill oil (dosage of 300 mg daily) significantly reduced CRP levels and arthritic symptoms such as pain, joint stiffness and functional impairment [10].

Cachexia is a complex and impairing loss of body weight observed in different diseases i.e. cancer, AIDS, congestive heart failure, etc., which is caused largely by a systemic inflammatory process. Cachexia worsens the outcome of the primary disease and a successful therapy is not yet available. Taylor et al. could confirm in a clinical trial that oral supplementation of cachectic tumor patients with 1.5 g/d MPL (Vitalipin^®^, see table 2) could alleviate tumor-associated weight loss while noticeably improving quality of life and physical activity [4]. These clinical results might further be explained by the inhibitory effects of PC on the pro-inflammatory TNFα-induced NFκB activation as shown *in vitro *by Treede *et al.*. NFκB causes activation of a number of pro-inflammatory genes as well as ATP-dependent ubiquitin proteasome formation, which leads to proteolysis of muscle cells, a pathway increased in cachectic wasting [11]. This means that the supplementation with MPLs during diseases causing cachexia could be recommended as supportive therapy to achieve stabilisation of the cachectic syndrome and therefore improve therapeutic results.

Another interesting therapeutic use of PL from marine sources is the application of Krill oil (see table 2) for the effective treatment of premenstrual syndrome (PMS). Since menstrual pain and cramps are supposed to be caused by n-6 FA mediated inflammation, the supplementation with n-3 FA could alleviate the mentioned symptoms. During a double-blind randomised clinical trial, krill oil was shown to be significantly more effective in reducing abdominal pain, swelling, breast tenderness and joint pain when compared to n-3 FAs from fish oil [12]. Furthermore, Sampalis et al. found an improvement in the emotional symptoms of PMS with the supplementation of krill oil, since brain PLs, which have a high content of DHA, are involved in brain function. Krill oil has the potential to modulate neurotransmitters and thereby, to positively affect the emotional and psychological symptoms in women with PMS. This means that the n-3 FAs bound to PLs are much more effective than FFAs in alleviating the mentioned symptoms, which could be explained by a much more efficient transport mechanism.

Non steroidal anti-inflammatory drugs (NSAIDs) inhibit the activity of the COX enzymes (COX-1 and -2), reducing the production of prostaglandins responsible for inducing pain symptoms and the related inflammatory reactions. Since NSAIDs (e.g. aspirin and ibuprofen) are not specific in inhibiting COX-2, the cyclooxygenase isoenzyme which is expressed under pathological conditions, they also affect the activity of the constitutively expressed COX-1 isoenzyme. COX-1 is - among other functions - in charge of regulating the production of natural mucus in the stomach to protect it from the secreted gastrointestinal (GI) acid and from pepsin hydrolysis during digestion, by catalyzing the production of PGE2 from AA, which reduces gastrointestinal acid production. This is the reason for the well known GI side effects of NSAIDs, since the GI protective effects of the mucus production are impaired by the unwanted inhibition of COX-1. However, a GI protective effect of PLs was shown with soybean PC (Phospholipon^®^100, see table 2), which reduced gastric mucosal lesions in rats after the treatment with NSAIDs (aspirin, indomethacin, phenylbutazone, diclofenac, piroxicam and sudoxicam). Co-administration of Phospholipon^®^100 with NSAID improved the drug tolerability and reduced the typical GI side effects, likely due to an increased production of cytoprotective mucosal PGE2 [13]. Since soy PC does not contain AA, the necessary AA for the production of PGE2 was probably formed by the conversion of linoleic acid (n-6 FA) to AA by the enzymes delta-6-desaturase, elongase and delta-5-desaturase. These GI-cytoprotective effects of PLs were also seen in patients presenting GI symptoms caused by a regular use of NSAIDs [14]. All patients were supplemented with Phospholipon^®^100 for 14 days resulting in ulcer healing as well as diminished upper abdominal pain. Another study by Dial et al. describes the beneficial effects of PC pretreatment (Phospholipon^®^90G, see table 2) in preventing LPS induced permeability changes of the GI tract in rats. This study shows that PC has protective effect not only due to supporting the PGE2-production [15].

Already in 1984 it has been found that (milk) PLs have a protective effect against gastric acid. An investigation in rats showed that treatment with milk reduced the ulcerating effects of intraluminal application of HCl. This effect was attributed to the concentration of di-palmitoyl-PC (DPPC), which is one of the major components of milk PLs [16]. During the following years the same group of researchers could substantiate their original findings with a large number of animal and human studies [17-19].

Besides the mentioned anti-inflammatory effects of GPLs in the GI tract, Stremmel et al. have found positive results of soybean PC (Sterpur P-30, see table 2) with regard to ulcerative colitis. One component of the colonic mucus is PC, arranged as lamellar layer and acting as mucosal defense [20]. The authors found a 70% decrease of mucosal PC in ulcerative colitis. In various clinical trials the authors found positive effects of the administration of delayed-release oral PC to patients with ulcerative colitis. They discussed that a physicochemically increased hydrophobicity of the intestinal lining could be responsible for the protective effects on preventing and healing inflammatory ulcerative colitis, since a defective PC layer contributes to inflammation; and second, PC has the ability to be integrated into the plasma membrane of enterocytes, modulating their signal transduction [21]. This has been shown for example in the study of Treede et al. where they found that PC was able to change the association between proteins and lipid microdomains, inhibiting thereby TNF-α-induced inflammation in Caco-2 cells [22].

In addition, PLs have shown to improve the pharmacokinetics of some drugs including NSAIDs. Lichtenberger et al. and Dial et al. have demonstrated soybean PC (formulation of NSAIDs with Phospholipon^®^90G, see table 2) to increase the anti-inflammatory and analgesic activity of NSAIDs in acute and chronic models of arthritis, by enhancing its transport and bioavailability [13,23-25]. Therefore, the administration of NSAIDs together with PLs could be an interesting attempt to not only enhance their analgesic activity, but also in diminishing the known side effects and reducing the risks caused by their regular use.

### Phospholipids and cancer

Several studies have described beneficial effects of PLs in tumor and metastasis inhibition. Some investigations have shown that cancer cell membranes acquire particular properties, which vary from those found in the differentiated progenitor cells. For example, the membrane of neoplastic cells showing the ability to metastasize have lost their adhesive characteristics as found in normal cells [26]. This enables cancer cells to dissociate from their surrounding (tumor) tissue and to migrate to other tissues or organs, causing tumor metastases. The membrane of breast and prostate cancer cells was shown to have a higher concentration of lipid rafts (areas with high cholesterol content) than their normal counterpart cells, which was associated with higher apoptotic sensitivity (regulated by its cholesterol content). Consequently, the regulation of the composition and density of lipid rafts could potentially alter cancer cell viability and metastatic behaviour [27].

A number of studies have investigated PLs for their ability to inhibit cancer growth. An *in vitro *study with hepatic cancer cell lines showed a dose dependent growth restraint when the cancer cells were cultured in the presence of soy and egg yolk PC (96.5% pure PC from soybeans and 99% pure PC from egg yolk) and menaquinone-4 (vitamin K2) [28]. Supplementation with PC and menaquinone-4 was also tested in rats, showing a clear suppression of nodule formation (precursors of hepatic cancer) and preneoplastic liver lesions in supplemented rats, compared to the control group. Although the effects were more significant when administering PC together with menaquinone-4, which act synergistically, PC alone also showed a statistically significant reduction of cancer cells via death ligands (i.e. Fas or TNF-α), thereby promoting apoptosis by the activation of caspase-8 and -3, resulting in PAP (poly(A)-polymerase) inhibition. Due to the interesting results, the authors are planning to examine these effects in a clinical trial, where a PC supplementation (900 mg/day of PC) will be used with the aim to prevent hepatocarcinogenesis in humans [28,29].

Beyond the above mentioned effects of administering NSAIDs together with PLs, i.e. the reduction of GI side effects and the improvement of analgesic activity, NSAIDs have also been shown to reduce growth of cancer cell lines [24]. Cancer patients commonly use NSAIDs for pain management on a regular basis, which causes GI side effects in most cases. Therefore, formulations of NSAIDs bound to PLs could effectively reduce bleeding and ulceration during pain therapy, while their additional effects in preventing cancer cell growth would be another advantage of this co-administration. To this regard, Dial et al. demonstrated that in colon cancer cell lines (SW-480, without expression of COX-2) the combination of soybean PC (Phospholipon^®^90G, see table 2) with NSAIDs, especially Ibuprofen-PC, had greater potency to inhibit cellular DNA synthesis than NSAIDs alone. Since SW-480 cells do not express COX-2, the result must be independent of the COX-2 inhibition and was shown to be related to an inhibition of phosphoinositol specific phospholipase C (PLC) and a subsequent suppression of protein kinase C, resulting in reduced cancer cell growth [24].

Moreover, the administration of marine PLs (PC extracted from squid meal and from starfish, both containing n-3 FAs) was shown to inhibit growth of chemically induced colon cancer *in vitro*. A significantly increased apoptosis rate was found in rats being fed with PC from squid (rich in DHA) and PC from starfish (rich in EPA), which was explained by an increased lipid peroxidation rate as a consequence of structural and functional shifts in the cellular membrane [30]. This was also observed after combining DHA or EPA (as free fatty acids or bound to PLs) with sodium butyrate (NaBt). In addition, they investigated the impact of other MPLs (PC together with PS extracted from starfish) as chemotherapeutic agent on Caco-2 tumors in vivo, showing a growth inhibition and an induction of cell differentiation. This effect was again more pronounced when adding NaBt to the PL due to an additional antiproliferative effect. The mechanism by which the n-3-FAs exert their inhibitory effect on tumor growth is still not completely understood. However, PLs must play an important role since FAs bound to PLs have a more pronounced effect when compared to the respective FFAs [31]. The results suggested that marine PC and PS carrying the n-3 FAs EPA and/or DHA can be used as colon cancer chemotherapy agents alone or in presence of NaBt [32].

Moreover, a mouse model was used to show that dietary sphingomyelin (SPM, see table 2) had a drastic preventive effect on colon cancer formation [33,34]. A possible mechanism of action of sphingolipids in suppressing colon carcinogenesis is the action of sphingolipid metabolites like sphingosine, sphingosine-phosphate and ceramide to induce apoptosis in a human adenoma cell line.

Recently, a strong antimetastatic effect of empty liposomes consisting only of hydrogenated PC and cholesterol was reported in two different orthotopic pancreatic tumor models in mice (AsPC1, LNCaP) while the growth of the primary tumors was not affected [35,36]. Investigating the underlying mode of action revealed that the PC degradation product, namely hydrogenated lysoPC, is most likely pivotal for the observed effect. After liposome accumulation in tumor tissues, lysoPC can be generated from PC by phospholipase A_2 _(PLA_2_) which is known to be highly expressed by numerous aggressive cancer cells, including pancreatic cancer cells [37]. Hydrogenated lysoPC was found to be rapidly taken up by the tumor cells *in vitro*, which leads to a remarkable increase of hydrogenated FAs in the cellular membranes and to a loss of their adhesion properties. Thus, adhesion of the tumor cells to endothelial cells and to platelets, both key factors in the process of metastasis, is strongly reduced and might therefore explain the antimetastatic effects of the PC containing liposomes. In a metastasis model using B16.F10 mouse melanoma cells, the pretreatment with hydrogenated lysoPC resulted in at least 50% inhibition of metastatic lesions in mice [38].

### Phospholipids in the regulation of blood lipid profiles and cardiovascular risks

The oral supplementation with dietary PLs has been investigated extensively in relation to blood lipid profiles and cardiovascular risks. Several authors reported a significant total cholesterol lowering effect of soybean PLs in patients with primary hyperlipidaemia [39-41]. This was not observed in patients with normal lipid levels, due to opposed HDL and LDL responses to supplementation. After 30 days of treatment, total cholesterol, LDL cholesterol as well as TGs were significantly decreased, while HDL cholesterol was significantly increased [42]. These effects were also described by Kirsten et al. in patients with diabetes mellitus (which have commonly pathological lipoprotein profiles) supplemented with polyenylphosphatidylcholine (PPC) over a 2 month period [43].

Patients with coronary artery disease and hypercholesterolemia resistant to low lipid diet and treated with Lovastatin^® ^normally achieve a reduction in TG, total cholesterol and LDL levels but simultaneously display an increased platelet activity, which is an independent risk factor for cardiovascular disease. Many studies reporting treatment with Lipostabil^® ^(soybean PLs, see table 2) in patients with coronary heart disease have suggested not only beneficial effects in reducing cholesterol levels by up to 50%, but also in preventing platelet aggregation [44-46]. For this reason, a combined therapy composed of lipid lowering medication and PLs could be a more helpful approach for the treatment of hypercholesterolemia, at the same time controlling platelet function [47]. Marine PLs (MPL) were also shown to have remarkable lipid lowering effects. A placebo-controlled, manufacturer-sponsored study with krill oil came to favorable results regarding the effect on blood lipid profiles in patients suffering from hyperlipidaemia [48]. Krill oil supplementation led to a significant reduction of total cholesterol, LDL and TG levels, while HDL increased significantly. Similar observations were made by Taylor et al., where a significant HDL increase could be seen in tumor patients after six weeks of MPL supplementation (Vitalipin^®^, see table 2), while total and LDL cholesterol decreased [4]. Since the supplementation with traditional fish oil (FAs bound to TG instead of PL) lowers only blood TG levels [49], but has no effect on LDL and HDL levels [50], PLs must be responsible for the observed beneficial changes of blood lipid profiles.

Similar effects to those of MPL on plasma lipid profiles were found for soybean PC. In a study with patients suffering from hypertension and obesity a dietary supplementation with sunflower oil in combination with soybean PC (unknown composition) was compared to sunflower oil alone. The PL supplementation was shown to reduce serum total cholesterol level, LDL, apo A1 (apolipoprotein A1), apoB and fibrinogen, consequently leading to a positive clinical outcome regarding hypertension [51,52]. Furthermore, animal studies performed by Wilson et al. demonstrated that the cholesterol lowering effect of a lipid lowering diet could be enhanced by the supplementation of soybean PC (Central Soya, see table 2) because it does not reduce plasma HDL levels, which are usually reduced during a lipid lowering diet [53]. They compared a diet containing soybean PC with a diet containing equivalent amounts of linoleate and choline, to find that the PC diet resulted in stronger hypocholesterolaemic effects. The analysed fatty streak area was significantly smaller in animals fed with the soybean PC supplementation, compared to those fed with the linoleate and choline diet.

High apoA1 levels have also been described to be protective against arteriosclerotic disease. Polichetti et al. investigated the effect of soybean PC supplementation (SPC Nutrition et Santé capsules, see table 2) on the apoA1 system in patients with type IIA hypercholesterolemia. They found that soybean PC increased significantly the level of apoA1, decreasing simultaneously apoA2 and apo E levels and stimulating therefore the reverse cholesterol transport, which contributes to reduced risk of arteriosclerosis [54].

Noh et al. have reported that sphingomyelin (SPM, unknown composition), a sphingosine phospholipid and major component of milk phospholipids as well as hydrogenated egg PC, has a significant effect on blood cholesterol levels [55,56]. The involved mechanism suggested by the authors is a similar effect to that of phytosterols, which may slow the rate of luminal lipolysis, micellar solubilisation and transfer of micellar lipids into the enterocytes. However, a clinical study investigating the lipid lowering effect of a SPM enriched butter milk formulation (Lacprodan^® ^PL-20, see table 2) containing 2-3 times more SPM than the normal dietary intake, did not show any significant decrease of either plasma lipids or lipoprotein levels [57].

Different mechanisms have been proposed and experimentally explored to explain the beneficial effects of GPLs on blood lipid profiles. In that context, the enzymatic activity of the blood lipid metabolism after oral or intravenous administration of PC has been investigated. The results show that PC causes a beneficial shift in blood lipid profiles, mostly reducing cholesterol levels, by altering the activities of important enzymes in lipid metabolism (see table 3: Enzymatic activity of the lipid metabolism after administration of PLs (adapted from Gundermann 1993 [58])).

**Table 3:** Enzymatic activity of the lipid metabolism after administration of PLs (adapted from Gundermann 1993 [58])

Enzyme	Activity after PL administration	Consequence
Lipoprotein lipase (LPL)	⇑	Acceleration of the breakdown of lipoproteins rich in TG, which results in a reduction of LDL and VLDL
Hepatic triglyceride lipase (HTGL)	⇑	

Acyl-cholesterol acyltransferase (ACAT)	⇓	Reduction of cholesterol ester deposition in cells

Cholesterol esterase	⇑	Hydrolysis of cholesterol esters in the cells. Free cholesterol can be transferred to the blood and incorporated into HDL by LCAT, which results in higher HDL-level
Lecithin cholesterol acyl transferase (LCAT)	⇑	

Other explanations for the effects of PL on blood lipid profiles are related more to the biophysical effects of PL, influencing the intestinal absorption of cholesterol and other lipids. In the intestine, PLs have emulsifier properties and the ability to form a fat-water emulsion with cholesterol and other lipids, forming vesicles or micelles. The cholesterol transport from the intestine into the enterocytes depends on the emulsification of the dietary fats with biliary secreted PC - or PC from the diet. In the presence of bile salts, vesicles are converted into micelles, which is ideal for lipid intestinal absorption [1]. PLs play an important role during lipid intestinal absorption by facilitating the formation of micelles (as PC and later as lysoPC which is formed from PC by pPLA_2_). Monomolecularly dissolved fat molecules, which are easily released from those micelles, are subject to uptake by enterocytes. It has been speculated that the addition of excess PL to the intestine by PC supplementation may lead to the formation of oversized micelles, preventing enzymes to reach micellar core content and reducing lipid and cholesterol absorption [1,59]. Moreover, intestinal PLs are also able to interact with the cellular membrane of enterocytes, reducing their cholesterol absorptive capacity [1]. Some authors have described that the degree of saturation and the length of FAs bound to PLs also controls the quantity of cholesterol absorbed in the intestine. The higher the degree of saturation and the longer the chain length of the FA, the less cholesterol is absorbed [55,56,60]. One possible explanation for this finding is the fact that PL carrying saturated FAs are poor substrates for pPLA_2_, therefore hindering the enzyme from accessing the micellar lipids (formed mainly of cholesterol, mono- and diglycerides, and coated with saturated PL) and in consequence impairing the cholesterol uptake [56].

The increase of HDL cholesterol in the blood after PL supplementation could be attributed to the fact that PLs can partially be absorbed intact by the intestine and are incorporated preferentially into HDL [2]. Furthermore, PLs are the substrate for the lecithin-cholesterol acyltransferase (LCAT), which catalyses the conversion of HDL_3 _to HDL_2_, thereby collecting cholesterol from peripheral tissues and increasing HDL levels [53].

Cholesterol lowering effects were also observed when administering phosphatidylinositol (PI) extracted from bovine liver (see table 2) to rabbits. The authors found that PI is primarily incorporated into HDL lipoproteins, which have direct contact to the cellular membranes of peripheral tissue. Via this pathway, PI has the ability to change membrane properties, influencing the intracellular signalling cascade and stimulating cholesterol efflux from peripheral cells due to an alteration of intracellular calcium levels. The discharged cholesterol is transported by HDL into the liver for biliary secretion (vascular cholesterol homeostasis), causing a significant cholesterol lowering effect [61]. Furthermore, it has been demonstrated that the increase of intracellular calcium levels promotes the catabolism of HMG-CoA reductase [62]. Due to the alteration of intracellular calcium levels by PI, it could be assumed that PI also has the ability to reduce cholesterol via this cellular homeostatic pathway [61].

High homocysteine concentrations have been described to be associated with a greater risk for developing cardiovascular disease. Homocysteine is a non-protein amino acid synthesized from methionine. Homocysteine can be recycled into methionine or converted into cysteine with B-vitamins acting as co-factors. Since betaine (synthesized from choline) has been shown to diminish plasma homocysteine concentrations [63-65], a choline supplementation could also contribute to the same effect. In a double-blind, placebo-controlled clinical trial Olthof et al. investigated the supplementation with soybean PC (PhosChol, see table 2) on homocysteine plasma concentrations in men with mildly elevated levels. They found that PC was able to reduce homocysteine levels, thus being able to reduce the risk of cardiovascular disease [66].

Independent of blood lipid profiles, PC has recently been correlated to the risk of cardiovascular disease through its metabolites [67]. The authors observed that intestinal micro flora metabolised PC to trimethylamine *N*-oxide (TMAO), which was considered to promote cardiovascular disease together with two other PC metabolites, betaine and choline. They found an up-regulation of macrophage foam cell formation, which promotes atherosclerosis, when TMAO, betaine and choline levels were increased in blood [67]. But, the conclusion that intake or supplementation of PC directly affects the risk for developing cardiovascular disease can not be drawn from this study. The correlation was observed with TMAO or choline alone, but no experiment correlated cardiovascular disease with supplementation of PC itself. Furthermore, each intestinal segment has different composition of the micro flora. The article does not describe in detail in which part of the intestine the feeding tube was placed.

The results reviewed here clearly show that PLs do affect blood lipid profiles in a beneficial way, and the clearest evidence was shown for marine PL containing predominantly EPA and DHA as well as for soybean PC. However, the mode of action has been not investigated thoroughly and more research is needed to clarify the mechanisms behind these positive effects.

### Phospholipids and neurological development/neurological disorders

Age related memory impairment represents a gradual, but physiologically normal deterioration of memory function, which affects virtually every human being. It is known that during aging the lipid composition of brain cells changes. The amount of polyunsaturated n-3 FAs (3-PUFA) in the brain tends to decrease with age, consequently membrane fluidity is decreased and cholinergic activities via retarded Na^+^- and Ca^+^-channels in the membranes are reduced, since they require PC and PUFAs for their excitability and neurotransmitter release [68]. One can assume that memory decline and the diminished learning abilities found in the elderly are consequences of a decreased quantity of PC and/or PUFAs in the brain tissue. PLs have been demonstrated to be important and effective carriers of essential PUFAs, e.g. docosahexaenoic acid (DHA) to the brain. Some authors have proven the preferential incorporation of DHA bound to lysoPC over free or TG bound DHA into the young rat brain [69,70]. For this reason, oral supplementation of PL carrying n-3 PUFAs (which is a special characteristic of PLs from marine origin) may play an important role, not only in the elderly, but also during pregnancy and infancy, where DHA provision into the brain is essential for neurological development. However, studies have shown that a supplementation with PL purified from pig brains, from egg yolk and from soybeans, which are rich in DHA, may improve learning abilities and visual function in age related impairment and during an n-3 PUFA deficiency. Supplementation could restore the ideal composition of PUFAs in the brain cell membrane, in which PLs have the potential of facilitating their transport [68,71,72]. Also, supplementation with dilinoeyl-PC (DLPC) in rats has shown to facilitate hippocampal synaptic transmission in the brain, improving thereby their spatial learning and memory abilities [73]. A further study investigated the effects of DLPC and palmitoyl-oleoyl-PC (POPC) in rats and in patients with cognitive disorders. Oral co-intake of DLPC and POPC resulted in better learning and memory abilities, and improvement of cognitive disorders [74].

Moreover, chronic alcohol consumption depletes PL in brain cell membranes, especially PC. It is discussed that alcohol supports the depletion of enzymatic and non enzymatic antioxidant systems (e.g. glutathione) from the cell membranes, which in turn leads to increased levels of lipid peroxidation (especially PUFA) in the brain, but also in other cells such as erythrocytes. Since the brain has a high PUFA content and few antioxidative systems compared to other tissues, it is extremely susceptible to lipid peroxidation, which is the initial step for the removal of a PL from the cell membrane. Thus, the cholesterol/PL ratio increases in cell membranes, leading to higher membrane rigidity and decreases cell deformability. On the other hand, acetaldehyde as product of the ethanol metabolism is able to bind to proteins forming acetaldehyde-adducts, which inhibit or damage enzymes and deplete the antioxidant status, augmenting the above mentioned effects, which could lead to serious degenerative modifications in the brain, resulting in neurological disorders. Jayaraman et al. have described the role of PC (Essentiale L, see table 2) as antioxidative agent in the treatment of alcohol induced brain changes. They could show that after ethanol application, PC administration prevented oxidative stress on erythrocytes (including the RBC membranes) and various brain regions in rats by restoring antioxidative mechanisms. Thus, the authors suggested PC as a useful antioxidant after alcohol ingestion [75].

Furthermore, PC might be a useful choline donator for the treatment of neurological diseases. During fetus development, choline is important for the brain tissue, since it serves on one hand as component of the cellular membrane and on the other hand as acetylcholine (Ach) precursor. It was demonstrated that rat pups showed better memory results when mothers were supplemented with choline during pregnancy. Choline had an influence on cellular proliferation, apoptosis and on epigenetic DNA properties, affecting brain development and thereby lifelong memory characteristics [76]. In a placebo controlled clinical trial, Ladd et al. found that the supplementation of PC (TwinLabs PC-55^II^, see table 2) in normal college students lead to an improvement in explicit memory due to increased choline supply and to improved cholinergic function [77].

Contrary to cognitive impairment, which could be a physiological normal deterioration, Alzheimer's disease is of different nature, generally irreversible and has a pathological background. An example regarding Alzheimer's disease shows that the membranes of affected brain cells have lower concentration of GPLs, i.e. ethanolamine plasmalogens^III^. The proposed mechanism behind the PL decrease is a higher activity of the plasmalogene-selective phospholipase A_2 _(PLA_2_), causing an increased release of free FAs and eicosanoids, which are coupled to lipid peroxidation and, most importantly, changing the membrane composition (membrane integrity, permeability and the structure of lipid microdomains) [78]. Since Alzheimer's disease are also linked to inflammatory processes, anti-inflammatory drugs might have the potential to slow its progression [79]. In this regards, Pandey et al. investigated the effects of (DLPC) on neuronal cells, finding reduced neuronal inflammatory activities through the inhibition of NFκB and MAPK [80]. All of these factors contribute to neurodegeneration in Alzheimer's disease, so that a supplementation with dietary GPLs could be effective in altering the cellular composition of brain cells and contributing to better therapeutic results. Other clinical studies have not found beneficial effects of PLs (especially PC) in Alzheimer's disease and since it is a complex pathology, more research is needed to clarify the inconsistent results.

Besides the mentioned effects of PC in improving memory and cognition, the highly specialised GPL, phosphatidylserine (PS), has an outstanding importance in the function of brain and neural cells. PS is able to revitalise memory, learning, concentration and even vocabulary skills - all those functions which have been found to be decreased with age. PS was originally extracted from bovine cortex, now it is also made from soya PC by enzymatic head group exchange.

In the last 25 years PS has been subject of a large number of randomised, double blind, placebo-controlled clinical trials. Together with more than 2.800 scientific papers about brain and neurological function, it is by far the best studied GPL for brain performance [81,82]. With aging, the sharpness of memory and cognition functions decreases, condition called Age Related Cognitive Decline (ARCD). These functions may lead to severe neurological disorders, not only in sick subjects, but also in clinically healthy individuals, which have difficulty in accepting the effects of cognition impairment [83]. A study of Crook et al. investigated short term memory function (primary parameter was the name/face acquisition and remembering the phone numbers) with the supplementation of PS (unknown composition). After just 3 weeks of PS supplementation they observed a statistically significant improvement of the physiological loss of cognition. In comparison to the control group, patients with PS supplementation had "rolled back the clock" of their cognitive age for 12 years in average [84]. Furthermore, a review paper described 9 double blind, placebo- controlled clinical trials investigating the outcome of PS treatment of a total of 1.224 patients affected by ARCD and/or early Alzheimer's Disease. It was shown that PS supplementation provided a strongly significant improvement in cognition and memory functions compared to the placebo group [85].

PS has further benefits regarding other brain activities like coping with stress and fighting depression. In young and healthy male patients the supplementation of PS showed to lower the production of stress hormones linked to strenuous exercise and eased stress-related mood symptoms [86]. Recent studies with soya PS supplementation showed stress lowering effects and improvements on concentration parameters in young and healthy subjects. Interestingly, the effects were more pronounced in patients with introverted personal behaviour [87].

Furthermore, supplementation with PS has been described to improve cognitive, neuropsychological and daily life performance through raising glucose levels in the brain. In patients with probable Alzheimer's disease, the brain metabolism was analysed with Positron Emission Tomography (PET) showing a threefold increase of glucose levels [88].

Another effect of PS is the stimulation of acetylcholine synthesis, which triggers the release of neurotransmitters from the vesicles to the synaptic gap. This effect may explain the reason of significant improvements of the physical performance in cycling tests after intravenous administration of brain cortex-derived PS (see table 2) [89].

The fact that the different FA composition of bovine derived PS or soya derived PS does not have any clinical relevance led to the hypothesis that naturally occurring PS found in milk GPLs could also have similar effects. Two clinical studies investigating the daily intake of a milk PL concentrate rich in PS on working memory, allostastic load^IV ^and acute stress response showed positive results. Compared to placebo-exposed individuals, there was a tendency to shorter reaction times in the working memory task, suggesting better performance in milk PL treated subjects. The two treatment groups did not significantly differ regarding their endocrine stress response. However, subjects in the PL-arm showed a blunted psychological stress response with a higher stress load. The results show beneficial effects on cognitive performance with the supplementation of milk PL, which is most probably attributed to its PS content. However, the benefits of milk PS may only be visible in chronically stressed subjects [90,91].

Improvements of memory, cognition and motility were also shown in patients with Parkinson's disease after supplementation with soybean PLs (formulation containing 25% PC), although no beneficial effects were observed regarding Parkinson's disease itself [92]. Studies with PC were also performed in other neurological diseases e.g. in patients with Friedreich's ataxia and patients with Gilles la Tourette's syndrome, but no relevant clinical results were observed [93-95].

During cerebral infarction the brain content of PLs may also decrease and contribute to the related complications. To this regard Shi et al. showed that supplementation with lecithin (unknown composition) after the onset of cerebral infarction showed effective results by preventing the decrease of brain PLs [96].

### Phospholipids and immunological function

The cholesterol/PL ratio (C/PL) in the cell membrane increases with age. These changes in the composition of cell membranes have consequences for their properties and functions. For example in lymphocytes an increase of the C/PL-ratio reduces their immunological function. Meczek et al. showed that the membrane viscosity of lymphocytes can be modulated by recovering the optimal C/PL-ratio. Therefore, it is expected that an increase of the PL content in lymphocyte cell membranes could restore the immunological function in the elderly [97]. An *in vitro *study by Rivnay et al. investigated the C/PL-ratio in lymphocytes after exposure to PC (Lecithin liposomes, unknown source). Lymphocytes of old mice had an increased C/PL-ratio in their membranes. Supplying the mice with the mentioned PC enhanced the proliferative response of lymphocytes. This effect was not significant in lymphocytes of young mice, supporting the theory that external PC (e.g. dietary PC) restores the optimal C/PL ratio in the affected lymphocyte membrane and probably in all other cells, too, maintaining their normal cellular functions [98].

The effect of dietary soy PC (Herbarium, see table 2) on the macrophage phagocytic capacity and on lymphocyte number in response to concanavalin A (ConA)^V ^stimulation was examined in vivo in rats. Lymphocyte number and macrophage phagocytic capacity were significantly improved with soy PC supplementation, indicating a modulatory positive effect of PC on the immune function [99].

In a further study, Jannace et al. investigated the effects of soy PC (unknown product) on phagocytosis, arachidonic acid (AA) concentrations and on neutrophil killing in healthy individuals. They found a significant improvement of polymorphonuclear leukocyte (PMNL) phagocytic and killing activity, and a significant increase in AA release in individuals fed with soybean PC, compared to individuals receiving placebo. The AA release indicated the ability of soy PC to change the membrane composition of PMNL, resulting in an improvement of its immunological functions. The effects must be attributed to PLs, since individuals fed with safflower or soybean oil (TG instead of PLs) did not show the same immunological benefits [100].

### Phospholipids and liver diseases

GPLs have been widely prescribed for the treatment of hepatic disorders including viral hepatitis and alcohol induced liver damage. A recent review by Gundermann et al. shows that essential PLs (purified extract of PPC derived from soybean) are indeed beneficial in the mentioned hepatic disorders. Although the use of essential PLs was characterized to be evidence-based medicine, more controlled clinical trials are required to determine its benefits [101]. The next paragraphs include some examples of the use of GPLs to treat liver disease.

During alcohol consumption the liver cells display reduced membrane PL levels. Many studies have shown that after supplementation with polyenylphosphatidylcholine (PPC, especially DLPC), the provided PL may be directly incorporated into the membrane of liver cells; normalising, among others, the activity of membrane-bound enzymes (i.e. phosphatidylethanolamine methyltransferase, PEMT^VI^) and reducing alcohol induced liver injury. For example, the study of Lieber et al. investigated the effect of soybean PPC supplementation (see table 2) during a 10 year period of chronic alcohol consumption in primates. In contrast to animals treated only with ethanol, animals treated with ethanol and soybean PPC showed reduced fibrinogenic liver damage and consequently had less cirrhosis development [102]. The mechanisms discussed could be attributed to the PPCs ability to prevent acetaldehyde-mediated collagen accumulation, thus preventing liver fibrosis [103]. This effect was also investigated in humans during a randomised placebo-controlled clinical trial continuing for almost 20 years, showing that PPC ameliorated liver disease in drinkers. Other positive properties of DLCP regarding alcohol induced liver damage are its ability to reduce hepatocyte apoptosis, as well as generation of TNF-α by Kupffer cells and lipid peroxidation [59]. The PC supplementation was shown to prevent not only the development of alcohol induced cirrhosis, but already the early liver changes induced by alcohol consumption (e.g. fatty liver and hyperlipidemia). Usually, alcohol intake induces an increase in plasma lipids, which are later transported to and accumulated in the liver (mainly TG and cholesterol ester) causing the alcohol induced hepatic damage [104]. Soybean DLPC supplementation (see table 2) was shown to prevent lipid accumulation in the liver, by reactivating the ethanol induced inhibition of mitochondrial FA oxidation, thereby reducing liver damage [104]. All the above mentioned effects were attributed to the whole PL, since the effects were not observed when supplementing FFA or the head group alone. Also, an animal study in rats demonstrated that PLs provided much better results in reducing liver TG levels when compared to a TG supplementation with almost the same FA composition as the PL supplementation [105].

In a clinical trial including patients with chronic alcohol consumption and alcohol induced liver damage, treatment with soybean PC (Lipostabil^®^, see table 2) was shown to be effective in improving liver related symptoms (e.g. cholestasis and icterus) while the hepatic antioxidant status was also restored [106].

Furthermore, PL supplementation was shown to be effective not only in improving alcohol related liver disease, but also after hepatic damage caused by toxins and virus infections. Some studies showed an amelioration of hepatic symptoms and an enhancement of liver functions with soybean PC supplementation in patients presenting non alcoholic liver damage [102,107]. Moreover, soybean PC (Essentiale^®^, see table 2) was given to patients presenting dramatic circulatory problems and simultaneous hepatic disorders, resulting in enhancement of hemodynamics, intrahepatic cholestasis, liver function and in the general lipid metabolism [108]. With regard to viral liver infections (hepatitis), a randomised clinical study was carried out in patients with hepatitis B or C. They received a combined therapy composed of the standard therapy interferon alpha (IFN) and either PPC or placebo. The results showed positive effects in patients suffering from hepatitis C, but not in patients presenting hepatitis B. The combined therapy of PPC and IFN improved the response rate of the standard therapy (IFN only) and the curative effect lasted longer compared to patients treated with IFN and placebo [109]. The fact that the observed effects were not seen in patients with hepatitis B could be attributed to the different pathophysiological cell damage of the two viral diseases.

Not only plant derived PLs, but also the supplementation with PL-rich dairy milk extract has shown to protect from liver damage by reducing total liver lipid, liver TG and total cholesterol. This was analysed by Wat et al. in mice fed with a high fat diet and PL-rich dairy milk extract (15% PE, 15% PC, 12% SPM, 8% PS, 4% PI and other ingredients). They could show that the expression of enzymes in charge of the hepatic FA synthesis was significantly decreased and that the bile acid production was also reduced, which might be due to a lower intestinal absorption of cholesterol. This study suggests that PLs from milk and dairy products have the potential to protect not only against cardiovascular events by reducing hyperlipidemia, but also to reduce hepatomegaly and hepatic steatosis [110].

## Conclusions and Discussion

This review provides an overview of the potential uses of GPLs as active ingredients for the treatment of different diseases. Many investigations revealed impressive health benefits of GPL supplementation without noticeable side effects.

It is impressive how GPLs, even though being "only" a food-component, are able to interact with the cellular membranes, changing their compositions (and probably their lipid microstructure and the lipid rafts) and thereby influencing a vast quantity of signalling processes and enzymatic activities. Since there have been many studies with remarkable effects performed during the past two decades, one could assume that GPLs are a "miracle drug". One explanation for the mentioned outstanding effects might be that our eating behaviour has changed during the last 1-2 centuries towards a suboptimal GPL-uptake, namely not enough GPLs and/or GPLs carrying the "wrong" FAs ("proinflammatory" n-6 FAs from i.e. meat and egg). The optimal n-3 to n-6 FAs ingestion is about 1:5. With the typical western diet the actual ratio is estimated to be 1:20, which is far below the recommendations. It has been shown that the supplementation with GPLs rich in n-3 FAs have the ability to compensate the "n-3 FA deficiency" in a more efficient way than other n-3 FA supplements (e.g. as TG or as free FAs) [100]. One could speculate that uptake of n-3 FA as GPLs are more efficiently incorporated into cellular membranes of different organs, thus affecting positively the membrane microstructure and its function. Thus, the benefits of GPLs reported in almost all studies could represent only the reestablishment of the normal GPL- and n-3 FAs requirements, so that GPLs as supplements could be very helpful in improving a great variety of health conditions. Since there are no side effects reported so far, GPLs have the potential to be consumed in a broader spectrum, that is, for many disorders at a time (as a regulatory mechanism by balancing the mentioned diet impairments regarding GPLs). Furthermore, a higher uptake of the right type of GPLs might also be recommended for healthy people as a prevention strategy to improve public health. This can be achieved by a regular uptake of GPLs as supplements and/or by preferential intake of food with sufficient and the "right" content of GPLs. Once more, it has become clear, that a balanced and healthy diet has positive effects in preventing and/or in stabilising/improving diseases, now increasing awareness about the potential importance of GPLs.

In contrast to the sometimes impressive effects of GPLs, the scientific explanation behind these effects is far from sufficient. The entire metabolism of GPLs has not yet been completely investigated and many questions remain to be answered. More research is needed to understand the role of GPLs after their ingestion and how they interact with the various processes of our complex metabolism. There are still many effects which remain to be discovered and results from larger clinical trials are eagerly awaited.

## List of abbreviations

AA: arachidonic acid; ARCD: age related cognitive decline; ATP: adenosine triphosphate; COX: cyclooxygenase; CRP: C-reactive protein; DGLA: dihomo-gamma-linolenic acid; DHA: docosahexaenoic acid; DLPC: dilinoleoyl- phosphatidylcholine; DPPC: di-palmitoyl-phosphatidylcholine; EPA: eicosapentaenoic acid; FA: fatty acid; FFA: free fatty acid; GI: gastrointestinal; GPL: glycerophospholipids; HDL: high density lipoprotein; HMG-CoA: hydroxy-methylglutaryl-coenzyme A; iNOS: nitric oxide synthase; IFN: interferon alpha; LCAT: lecithin-cholesterol-acyl-tranferase; LDL: low density lipoprotein; LPS: lipopolysaccharide; MAPK: mitogen-activated protein kinases; MPL: Marine phospholipids; NaBt: sodium butyrate; NFκB: nuclear factor kappa-light-chain-enhancer of activated B cells; NSAIDs: non steroidal anti-inflammatory drugs; PC: phosphatidylcholine; PE: phosphatidylethanolamine; PEMT: phosphatidylethanolamine methyltransferase; PET: positron emission tomography; PGE: prostaglandin E; PI: phosphatidylinositol; PL: phospholipid; PLC: phospholipase C; PMNL: polymorphonuclear leukocyte; PMS: premenstrual syndrome; PPC: polyenylphosphatidylcholine; pPLA_2_: pancreatic phospholipase A_2_; PS: phosphatidylserine; POPC: palmitoyl-oleoyl-phosphatidylcholine; PUFA: polyunsaturated fatty acid; RBC: red blood cells; SPM: sphingomyelin; TG: triglyceride; TMAO: trimethylamine N-oxide; TNFα: tumor necrosis factor-alpha; VLDL: very low density lipoprotein.

## Competing interests

The authors declare that they have no competing interests.

## Authors' contributions

All authors have made substantial contributions to this work. DK designed and drafted the manuscript after literature research. LAT supported literature research, drafting and final corrections of the manuscript. MS contributed to the manuscript regarding the health effects of milk phospholipids and those from phosphatidylserine. UM coordinated and contributed to drafting of the manuscript. All authors read and approved the final manuscript.

## Authors' information

DK is a nutritional scientist and PhD student at the Tumor Biology Center Freiburg. LAT is a pharmacist, working at the Pharmacy of the University Hospital Heidelberg.

MS is a chemist, founder and owner of Lecithos Consulting Jesteburg.

UM is a chemist, working at the Tumor Biology Center Freiburg as head of the working group "Lipids and Liposomes". He also gives lectures at the University of Freiburg.

## Endnotes

^I ^The *sn *nomenclature is based on the *stereospecific numbering *of the carbon atoms of a glycerol molecule. It is used when a molecule has a chiral center (a carbon atom which has no symmetry), in this case, the glycerol backbone of a PL.

^II ^1 g of TwinLab PC-55 supplies 0,15 g of choline.

^III ^Plasmalogens are ether lipids composed of glycerol, a vinyl residue, a FA and a PL.

^IV ^Allostatic load is a hightened neural response as a consequence to repeated stress.

^V ^ConA was used for activating lymphocytes

^VI ^The PEMT enzyme is in charge of regenerating hepatic PC, which is normally decreased during alcoholic liver damage
